# A Methodology for Evaluating Operator Usage of Machine Learning Recommendations for Power Grid Contingency Analysis

**DOI:** 10.3389/fdata.2022.897295

**Published:** 2022-06-14

**Authors:** John Wenskovitch, Brett Jefferson, Alexander Anderson, Jessica Baweja, Danielle Ciesielski, Corey Fallon

**Affiliations:** ^1^Pacific Northwest National Laboratory, National Security Directorate, Richland, WA, United States; ^2^Pacific Northwest National Laboratory, Energy and Environment Directorate, Richland, WA, United States

**Keywords:** human-machine teaming, trust evaluation, cognitive load, power grid, contingency analysis

## Abstract

This work presents the application of a methodology to measure domain expert trust and workload, elicit feedback, and understand the technological usability and impact when a machine learning assistant is introduced into contingency analysis for real-time power grid simulation. The goal of this framework is to rapidly collect and analyze a broad variety of human factors data in order to accelerate the development and evaluation loop for deploying machine learning applications. We describe our methodology and analysis, and we discuss insights gained from a pilot participant about the current usability state of an early technology readiness level (TRL) artificial neural network (ANN) recommender.

## 1. Introduction

The field of power systems represents an exemplar for domains that struggle to incorporate new technologies. Challenges with control room work culture tend to stifle technological innovation and prevent the evolution of technology (Kennedy, 1995; Von Meier, 1999; Nisar et al., 2013). To an extent, this can be expected, as power grid operators are responsible for critical services, managing transmission and distribution of electricity to cities, residences, and other industrial loads. Their complex jobs involve maintaining situational awareness of their area of responsibility (AOR), monitoring voltage and frequency, balancing generation and load, responding to outages, and responding to emergency scenarios. With high workload and high stakes, operators must have reliable technology that doesn't interrupt the speed or quality of work. However, grid decarbonization, adoption of smart grid technologies, and displacement of traditional generation by distributed renewables will soon disrupt current power systems operational paradigms. As a result, a new generation of advanced power applications will be needed to ensure reliable, resilient, robust, safe, and economic operations.

Machine learning advances can make it possible to aid operators and engineers in critical action decisions, particularly when time pressure is involved. A notable implementation of machine assistance is accurate forecasting of total system load as well as solar and wind expectations for generation scheduling and energy market pricing (Lei et al., 2009; Makarov et al., 2011; Almalaq and Edwards, 2017; Wang et al., 2019; Lai et al., 2020; Vanting et al., 2021). Despite the forecasting benefits that machine learning can provide, a variety of reasons limit the adoption of these tools by electric utilities (see Section 2.1). One of the largest hurdles is that developers of future systems must consider not only the technical aspects of machine learning (architecture of the algorithm, training/ validation data quality, etc.), but also incorporate a wide range of operational considerations and, ultimately, measure whether new systems are usable by power system operators. These include consideration of both technology readiness levels (TRL) (Mankins, 1995) and human readiness levels (HRL) (See et al., 2019), adherence to existing operational procedures and increased workload during the adjustment process (Ludwick and Doucette, 2009), as well as the impact of the tool on human-machine trust (Hoff and Bashir, 2015), user autonomy (BenMessaoud et al., 2011), operator situational awareness, and grid performance. As a result, the current tool deployment process in power grid control rooms is drawn out and deliberate.

We focus in this work on evaluating the introduction of machine learning to address the problem of contingency analysis in power systems. Contingency analysis (CA) is a process used to identify potential violations of equipment and operational limits due to unexpected loss of any single piece of physical equipment. Performing this analysis allows operators to identify the best course(s) of action to keep the power grid operational. Operators currently rely on predetermined procedures and their own experience to resolve contingencies in the power grid. This study evaluates the readiness and usability of a recommendation system called ACAT (Artificial Intelligence [AI]-Based Contingency Action Tool). This system makes use of an artificial neural network (ANN) to recommend contingency mitigation actions to operators in order to quickly address potential violations.

To emphasize the importance of operator workflows, human expertise, and how advancement on the HRL scale can facilitate TRL advancement, our research team considered an early stage TRL algorithm that was designed to provide recommendations to control room operators and engineers for mitigating potential power system violations. The disconnect between technology developers and end-users is a living obstacle in getting well-meaning technology in the hands of mission-critical personnel that have to work with these proposed tools and systems on a daily basis. Our work aims to provide an example of how a human factors study can be conducted with an end-user to provide actionable feedback to developers. We seek to accelerate the development and evaluation loop inherent to these development cycles by rapidly collecting information from domain experts that addresses these operational procedures. This includes the acquisition of objective, subjective, and qualitative feedback measures to address cognitive load, trust, human readiness, and the technological readiness, usability, and impact of a novel system. The captured data are aggregated and analyzed to report back on the current state of the system under evaluation.

In particular, we contribute:

- A methodology that measures domain expert trust, cognitive load, and technological usability of recommender systems, with the goal of rapidly acquiring and analyzing objective, subjective, and qualitative measures.
- Results of the application of this methodology to an early-stage tool designed to provide contingency mitigation recommendations to power grid operators.
- Insights gained from observing user interactions under individual scenario conditions and difficulties.

The methodology applied here provide a framework for developers and human factors experts evaluating recommender systems, with a particular focus on how humans respond to and rely upon the provided recommendations. Researchers can then apply this methodology when integrating those systems into a workflow. The methodology proposed should help to identifying limitations and shortcomings in the system or challenges in its integration into human operations. This will support improvement of the system under development and accelerate the integration of the system into operations.

## 2. Related Work

In this section, we discuss existing work in three topics related to this research. We briefly review common barriers to technology adoption (Section 2.1), we provide an overview of common terminology and discuss active research in human-machine trust (Section 2.2), and we describe the usage of heart rate variability as a proxy for understanding cognitive load (Section 2.3).

### 2.1. Barriers to Technology Adoption

From the many barriers to the adoption of new technology identified by Rogers (1962), we focus our discussion here on two relevant categories: barriers caused by the technology itself (TRL) and barriers caused by the user's ability to use the technology effectively (HRL). From cloud computing (Zhang et al., 2021) to smart grid technologies (Dedrick et al., 2015) to system and information security (Schoenmakers, 2013), the adoption of technology into control rooms encounters many of these barriers as well as others.

Characteristics that are inherent to the technology itself often are often the result of flaws with the system, including unreliability, failures, malfunctions, and low accuracy (Butler and Sellbom, 2002; Al Farsi and West, 2006; Kemper et al., 2006; Randeree, 2007; Schaeffer et al., 2013). Beyond these direct issues lie other problems that may result from the deployment of new technology, including information flow disruption (Poon et al., 2004; DePhillips, 2007; Schaeffer et al., 2013; Plaete et al., 2015), issues with data continuity (Ross, 2009; May et al., 2011), and issues with data standardization and interoperability (Ludwick and Doucette, 2009; Castillo et al., 2010; Lycett et al., 2014; Agalgaonkar et al., 2016).

Barriers related to the effective utilization of technology by users are also varied. General challenges with system usability and related difficulties in use are often a significant concern of new adopters (Venkatesh, 2000; Lærum et al., 2001; Miller and Sim, 2004; Meade et al., 2009; Holden, 2011). Related to these challenges are concerns about a loss of productivity while integrating a new tool into a workflow (Snoeyink and Ertmer, 2001; Meinert, 2005), as well as the accompanying increased workload that is inherent to the adjustment process (Reardon and Davidson, 2007; Simon et al., 2007; Ludwick and Doucette, 2009; Rao et al., 2011). When thinking specifically about the introduction of machine intelligence, loss of user autonomy and agency is also a concern raised by users (Ross, 2009; BenMessaoud et al., 2011; Wenskovitch et al., 2021), as is a lack of trust in conclusions reached by the machine (discussed in the next subsection).

### 2.2. Human-Machine Trust

A critical consideration for introducing automation into an established process is ensuring that the human maintains an appropriate degree of trust in the automation (Wenskovitch and North, 2020). Lee and See 2004 define trust as “the belief that a technology will help an individual accomplish his/her goals in situations of high uncertainty and complexity.” Lyons and Stokes argue that trust also captures one's willingness to be vulnerable to the machine entity (Lyons and Stokes, 2012), while Onnasch et al. (2014) note that the consequences for automation errors are the most severe when the systems are exhibiting the highest levels of automation. Trust factors can be measured by the performance of the human, the machine, and/or the team as a whole (Damacharla et al., 2018). These metrics fall into a variety of categories, such as measuring performance, safety, and efficiency. They can also be measured either subjectively or objectively.

Trust as an overall concept consists of three variable layers, identified by Hoff and Bashir (2015). *Dispositional* trust refers to the general tendency to trust automation, independent of both context and of specific systems. *Situational* trust depends on the current circumstances, exploring the development of trust with the current system and problem. *Learned* trust is an operator's evaluation of a system over time, drawn both from past experiences as well as the current interaction. Fostering the appropriate amount of trust in automation is a continuous challenge that optimizes the tradeoff between the capabilities of the system and the user's perception of those capabilities (Lee and See, 2004). There are penalties for both overtrust and undertrust in the capabilities of a system: *misuse* or *overtrust* refers to failures that occur when people inadvertently violate critical assumptions and rely on automation inappropriately, whereas *disuse* or *distrust* refers to failures that occur when people reject the capabilities of automation.

There are a number of antecedents of trust that can be utilized to foster additional trust in a system. *Familiarity* is perhaps the most obvious trust factor; a user who has a substantial amount of contact with automation will have more time to develop trust in the automation, understanding how and why it operates in the way that it does. Hoff and Bashir (2015) present a literature review on trust in automation, noting the role of familiarity and learned trust. Both *predictability* and *reliability* also play important roles; working with an automation that is predictable (Lee and See, 2004) and reliable (Hancock et al., 2011) enhances trust with the system. If a system can predict upcoming difficulties and/or provide *proactive assistance* without prompting by the analyst (and that assistance is dependable), the user can develop additional trust in the system (Ho et al., 2017). Finally, *transparency* provides a significant impact to trust in automation, including transparency of decision logic (Lyons et al., 2016b; Sadler et al., 2016; Wang et al., 2021), the intent that underlies actions taken (Lyons, 2013; Lyons et al., 2016a), and current system state (Mercado et al., 2016). The research presented in this study builds on Madsen and Gregor's Trust Questionnaire (Madsen and Gregor, 2000), described in further detail in Section 3.3.

Trust in automation remains a very active topic of research, exploring methods of enhancing trust, recognizing and addressing known challenges, and proposing a significant future research agenda. McDermott et al. (2018) provide a thorough guidebook that is designed to help system developers who are working to design trustworthy machine teammates, while Smith (2019) proposes a checklist and agreement framework for human-machine teams. Paul et al. (2019) present a research agenda that is specific to trust in human-machine teams for cybersecurity operations, identifying a need for better insight, context, and explainability in human-machine teams in this space. Several recent research endeavors have focused on producing guidelines for implementing artificial intelligence (AI) technologies into current workflows, as well providing advice for interacting with AI. The extensive guidelines produced by Amershi et al. (2019) include initial planning and overarching systems guidelines, guidelines that govern interactions, guidelines for handling when the AI is wrong, and guidelines that govern behavior with the system over time. Extending this work, Nushi et al. (2020) build a case for how to best apply these guidelines, including setting the right expectations for users, mitigating bias, and supporting interaction experiences over time.

### 2.3. Cognitive Load and Heart Rate Variability

Another consideration for integrating automation into a workflow is the effect on the cognitive load level of the human. If using the new system increases the difficulty of the task, then overall performance could be impacted by higher cognitive load, or the amount of information that is currently being processed within working memory (Sweller et al., 1998).

Like trust, cognitive load can be measured by subjective or objective means. Subjective measures are typically evaluated by survey or debriefing following a task, and they include an evaluation of perceived difficulty or mental effort (DeLeeuw and Mayer, 2008). A frequently-used survey is the NASA Task Load Index (TLX), a survey that evaluates workload subjectively by asking a user about the mental demand, physical demand, temporal demand, performance, effort, and frustration level when completing a task (Hart and Staveland, 1988). Common objective measurements for cognitive load are often performance-based, including response time and number of errors (Sweller et al., 1998).

Another quantitative measure of cognitive load is heart rate variability (HRV). HRV measures variation in time between heartbeats, or more precisely the variation in recorded intervals between R spikes, the peaks of an electrocardiogram wave. Measuring this variability in heart rate consistently shows that when mental workload is increased, HRV decreases (Aasman et al., 1987; Porges, 1992; Sweller et al., 1998; Cinaz et al., 2013). In contrast, studies that measure only the heart rate rather than the variability have found both increases (Harris et al., 1990; Mulder, 1992; Wilson, 1993; Stuiver and Mulder, 2014) and decreases (Casali and Wierwille, 1983; Veltman and Gaillard, 1996) in heart rate when cognitive load is increased. A benefit to using HRV as a means of measuring cognitive load is the unobtrusive nature of many of the devices used to acquire physiological measures, though this is coupled with the clear limitation of requiring specialized hardware. Kramer (2020) reviews how a variety of biometric measures can be obtained and measured through these sensors and devices, quickly detecting phasic shifts in mental workload.

## 3. Methodology Components

In this section, we describe the components to the methodology that are employed to measure the impact of a new system on domain expert trust, cognitive load, and technological usability. The methodology and components are generalizable, although some of the particular metrics here are specific to the power systems domain. In Section 4, we describe the details of an experiment that demonstrates this methodology with an ideal user. This methodology incorporates the rapid acquisition of objective (Sections 3.1, 3.2), subjective (Section 3.3), and qualitative (Section 3.4) feedback from a participant. Table 1 provides an overview of the data, measurements, tools, and processing for each section of this methodology.

**Table 1:** Overview of the data, measure, tools, and processing for each component of the methodology.

**Data**	**Measurement**	**Tools required**	**Processing method**
HRV	Cognitive load	Zephyr bioharness	Neurokit2, custom code
Logs	Performance	PowerWorld	Custom code
Survey	Trust	Modified madsen and gregor	Statistical software
Interview	Insight	Survey and Conversation	Coding of responses within the research team

### 3.1. Heart Rate Variability

To capture the data needed to interpret heart rate variability (HRV), participants are fitted with a Zephyr bioharness^1^. This commercial device is capable of measuring six inputs and reporting more than 20 biometrics. Due to the demonstrated connection between increases in cognitive load and decreases in HRV (see Section 2.3), we use HRV as one proxy for understanding the cognitive load of a participant throughout the duration of an experimental trial. We also collect TLX data from each participant as a subjective measure of workload (see Section 3.3). The raw electrocardiogram (ECG) data are processed using NeuroKit2 (Makowski et al., 2021), a Python library for neurophysical signal processing, to extract a clean R-R interval (distance between the characteristic tall peaks found on the ECG) signal. Standard computations of ECG data include:

1. **SDNN:** Measured in milliseconds, this is the standard deviation of N-N interbeat intervals that have been filtered and artifacts have been removed.
2. **RMSSD:** Measured in milliseconds, this is the root mean square of successive R-R interval differences. R-R intervals are interbeat intervals between all successive heartbeats.
3. **LFn:** This is the ratio of the low frequency power (0.04–0.15 Hz) in the ECG frequency-domain to the total power.
4. **HFn:** This is the ratio of the low frequency power (0.15–0.4 Hz) in the ECG frequency-domain to the total power.
5. **LF/HF ratio:** This is the ratio of LF power to HF power.

There are a number of reviews that compare and contrast these metrics as measures of mental workload (Shaffer and Ginsberg, 2017). For short-term ECG recordings these metrics show the most promise for capturing workload consistently over the course of an experiment. Provided we required a more objective means to measure cognitive load, while balancing the ease of deploying sensors, other means of measuring workload (namely EEG and ERPs) were not used.

### 3.2. Performance Scoring

Performance for a given task is typically determined based on behavioral metrics and task relevant measures. When it comes to preventative actions, determining ground truth is a major challenge to measuring performance and impact. Part of the complication is that there is not just one way to mitigate potential violations and plan for contingencies.

When deploying the action recommendation algorithm, the research team decided to measure performance of operator actions through a mixture of well-studied behavioral markers and domain-specific measures. Below is a list of those metrics along with descriptions.

1. **Completion time:** This is a measure of time that starts with the time that the contingency is introduced to the participant until the time the mitigating plan is implemented. In control room environments, solving contingencies in a timely manner is key.
2. **Action Penalty:** This is a measure of overall system changes implemented in order to solve contingencies. Discrete actions such as switching shunts or turning on and off generators are counted. Discrete actions are combined with continuous actions such as absolute generation changes to provide an overall score. This penalty score is derived from existing guidelines surrounding operator impact on power grid systems (Anderson et al., 2022).
3. **System Penalty:** This measure complements action penalty and measures the degree to which the current state of the system violates operating limits. Each unit component of the power system has defined operating limits. This measure is the simple sum of the exceedance or amount under operating limits each component in the grid is currently operating. We use this metric as an objective function for performance, in that this is what the operator should be optimizing during a given trial (Anderson et al., 2022).

For real-world deployment, assistance algorithms are often engineered to obey strict rules about financial considerations, safety considerations, and operating limits. As an early stage TRL algorithm, ACAT development has been focused on providing meaningful and accurate feedback to operators. Our goal was to determine a set of useful metrics to aid in not only accurately describing objective performance, but also metrics that are useful to developers. While completion time provides a meaningful and often-used human factors measure, action and system penalties provide accuracy measures that could be useful for ACAT development. This is an example of how one might consider incorporating domain-relevant metrics with AI-relevant metrics in a usability assessment.

### 3.3. Surveys

After each trial, participants receive a modified version of Madsen and Gregor's Trust Questionnaire (Madsen and Gregor, 2000). This questionnaire measures several constructs that underlie trust in a system. These constructs can be divided into Cognitive-Based trust components (e.g., system understandability, technical accuracy, and reliability) and Emotion-Based trust components (e.g., faith and personal attachment to the system). See Table 2 for a description of each construct. With respect to the Cognitive trust components, it is important to note that this survey measures *perceived* reliability and accuracy, which may differ from the actual system performance. For each item, participants are asked to rate their agreement with a statement. Participant agreement is measured on a five-point Likert-scale with anchors ranging from 1 (Strongly Disagree) to 5 (Strongly Agree).

**Table 2:** The descriptions of each construct measured in our trust questionnaire.

**Construct**	**Description**	**Component**
Reliable	The system performs consistently.	Cognitive-based trust
Technical competence	The system performances accurately and correctly.	
Understandable	A user can understand how the system works and predict its performance.	
Faith	Belief that the system can perform well in the future, even in situations when it is untested.	Emotion-based trust
Personal attachment	A strong preference for working with the system.	

Two modified versions of the questionnaire are administered. One version is designed to measure trust in ACAT. For this version the language was modified to address ACAT specifically. For example, the item from the original questionnaire “The system analyzes problems consistently” is changed to “ACAT analyzes CA violation problems consistently.”

In the control condition of this experiment, participants did not use ACAT and instead rely completely on their operational procedures. A second version of the trust questionnaire is modified to assess trust in the operations procedures used during the control condition. For this version, the language is modified to address operations procedures specifically. For example, the item from the original questionnaire “The system analyzes problems consistently” was changed to “My operation procedures help me to analyze CA violation problems consistently.”

After each trial, participants also received the NASA Task-load Index (TLX). This self-report measure assesses a person's workload on six dimensions (Hart and Staveland, 1988). Three dimensions require one to consider the demands imposed on them by the task or environment. These dimensions include mental, physical and temporal demands. The other three dimensions ask participants to assess their own ability to manage these demands. These dimensions include self-rated performance, effort and frustration. Each dimension is measured by one item and the items are phrased as questions. Rubio et al. (2004) found support for the measures validity demonstrating high correlations (0.97 and 0.98) with other self-report measures of workload.

Participants rated their workload on each dimension using a six-point Likert scale. In our study, participants were asked to answer each question considering their workload during the scenario trial they just completed. For example, one item asks participants to consider “How mentally demanding was the trial?”

### 3.4. Semi-structured Interview Feedback

During each trial, we collect qualitative feedback as the participant comments on their actions and describes their approach toward mitigating a contingency. In order to maintain their focus on exploring the scenario and to enable us to accurately measure cognitive load, participants are not asked to follow any think-aloud protocol. Instead, we capture any spontaneous commentary as the participant works through each scenario. After the participant submits their final solution and begins work on the post-trial survey, we conduct a brief semi-structured interview, gathering additional feedback on the technological readiness of the system. A similar semi-structured debrief is also conducted at the end of the experiment, capturing the overarching thoughts and opinions of the participant after experiencing the ACAT system in a variety of scenario conditions.

## 4. Experimental Design

In this section, we describe the parameters of our experiment to evaluate the current usability state of the ACAT recommender tool. We describe the control room environment (Section 4.1) and the software (Section 4.2) available to the participant, summarize the experimental procedures (Section 4.3), provide a reference overview of the scenarios used in the experiment (Section 4.4), and present an example scenario that is representative of the contingencies that the participant is resolving in this study (Section 4.5).

### 4.1. Control Room

**Figure 1:**
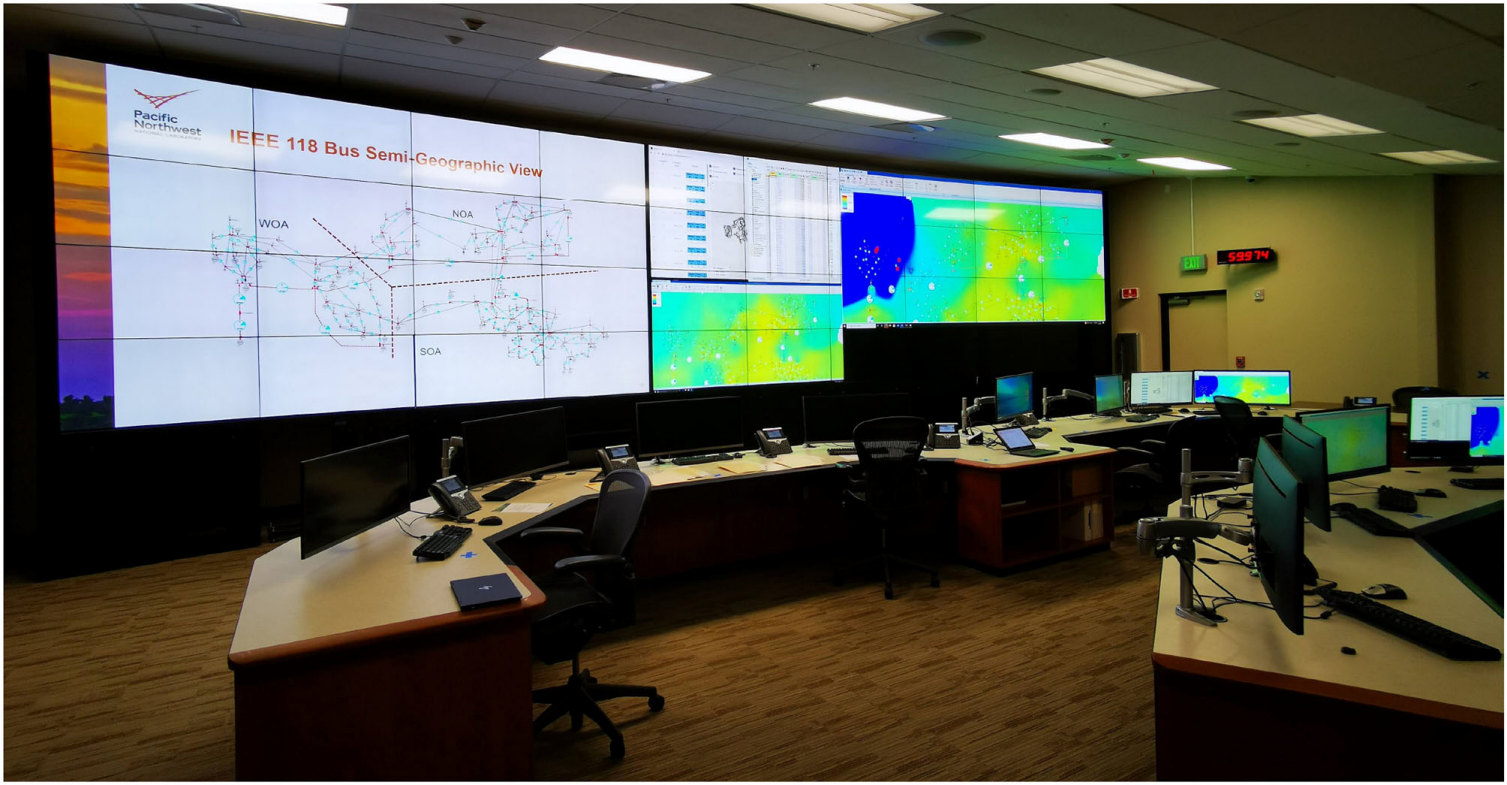
A photo of the electricity infrastructure operations center (EIOC) west control room at pacific northwest national laboratory (PNNL). On screen are a geographical schematic of the IEEE 118 Bus network, several instances of the PowerWorld Simulator, and the ACAT recommender.

The experiment was conducted in the Electricity Infrastructure Operations Center (EIOC) West Control Room at Pacific Northwest National Laboratory (PNNL), shown in Figure 1. The EIOC provides a realistic replica of a utility control room for transmission and distribution system operations. The control room includes 16 operator consoles arranged at three control desks, all of which are connected to a dedicated network and server enclave. At the front of the room is a large video wall system consisting of 40 individual monitors. The individual operator consoles and control desks can be configured to enable role-play between multiple participants serving as various North American Electric Reliability Corporation (NERC) functional entities, including reliability coordinator, balancing authority, transmission operator, distribution operator, and generation facility operator. This simulated control room environment provides opportunities for the evaluation of new systems, experiments designed to understand human factors, and realistic training scenarios. Due to COVID safety considerations and restrictions on room capacity, we focus this study on the interactions between a participant (rather than a full team of operators) and the ACAT recommender, seeking human subjects with substantial expertise in real-time contingency analysis and control room operational procedures.

### 4.2. Software

The participant in this study made use of two software systems during the experiment. Participant interactions were performed within PowerWorld Simulator, a commonly-used program within control room environments for exploring the effects of changes to the power grid. ACAT, the system under review in this study, is a recommendation system intended to direct operators toward optimal solutions for resolving contingencies.

#### 4.2.1. PowerWorld

PowerWorld Simulator^2^ is a commercially available software package used by utilities and researchers to perform common analysis and simulation tasks for high voltage power systems on a time frame ranging from several minutes to several days. It is able to import model files from a wide variety of other software packages and display them in an intuitive graphical user interface.

For the experiment, the participant was given three PowerWorld displays (seen in Figure 1) containing a tabular summary of equipment statuses, a one-line diagram with a semi-geographic view of the power grid, and a tabular summary of contingency analysis results. Equipment violations to be resolved by the participant were highlighted in the tabular display as well as with pie chart flow indicators on the one-line diagram.

Additionally, a custom logging script was written to record the actions taken by the participant in PowerWorld over the course of the experiment. This log file contained records of the particular piece of equipment operated by the participant (generator, line, shunt capacitor, etc.), new setting values for the equipment, and timestamps logging when the participant updated the power flow solution and contingency analysis results.

#### 4.2.2. ACAT

ACAT is the technology under evaluation in this study. At the time of this study, ACAT has a Technology Readiness Level (TRL) of 3 (on a 9-point scale) due to the fact that it had been trained to consider line and transformer outages on a single power system model with a fixed topology (Mankins, 1995). A TRL of 3 means that the technology demonstrates some core ability to address concepts of interest experimentally and analytically, but hasn't demonstrated that capability in the applied environment or in a laboratory environment. ACAT serves as a recommendation provider, displaying effective corrective action recommendations to remediate real-time contingency analysis (RTCA) violations. The system itself makes use of an artificial neural network and a semi-supervised corrective action algorithm (Chen et al., 2019). A large number of simulation base cases are generated that are unique to a specific grid, varying load and generation to represent a wide range of operating conditions (Chen et al., 2016). These simulation base cases are then passed to a contingency analysis tool (Huang et al., 2009) to identify violations and evaluate their severity. The ANN is then trained on the simulation base cases and accompanying violations. Currently, ACAT is a PNNL developed tool that is not publicly available. However, there is active research in developing prototype tools to aid control room operators in decision-making and to that end, the ACAT tool serves as a specific instance of a recommender system.

Solutions to new contingencies fed into the model are generated via the corrective action algorithm. This algorithm uses an iterative process to compare known stable system states to the load and generation profile under evaluation, developing a mitigation procedure to transition from a state with a violation to one without. This mitigation procedure is validated by the ANN to identify any potential follow-up violations that may result from applying the solution. Following this validation step, a sequence of control actions is then displayed to the operator as a recommended procedure for resolving the contingency. Recommendations generated by ACAT take the form of text on a large monitor in the control room, and they identify substations on the 118-bus system and what actions (shedding load, redistributing power, etc.) should be taken. The recommendations span the general class of actions that human operators can take as corrective actions. Operators are not required to act on these recommendations but can.

### 4.3. Experiment Procedures

Rather than collecting a team of participants to fill the collection of control room roles in a normal operating environment (e.g., a reliability coordinator, a balancing authority, and a transmission operator), this research focused on the interaction between a single participant and the ACAT recommender. We made this determination to maintain focus on interactions with the ACAT recommender, allowing us to better measure performance, trust, and the operator's real-time opinions about the quality of the tool and its recommendations during trials when ACAT was present. During each trial, the participant was responsible for identifying the contingency analysis violation, determining the proper mitigating actions, and implementing those actions in the PowerWorld simulation. The workflow deployed in this experiment is modeled off of actual workflows for power system engineers. The general steps include presentation of a scenario, an exploratory solving phase, a final solution implementation, and completing a post-scenario survey.

Two adjacent consoles were configured with two instances of the PowerWorld power system simulation software. The left console was set up as the simulated real-world Supervisory Control and Data Acquisition (SCADA) interface and one-line diagram. The right console was configured as a “study-mode” session, with two monitors containing tabular displays and one-line diagram from ACAT and PowerWorld. In the performance results presented in Section 5.2, these are labeled as “SCADA” and “study-mode” respectively. A paper copy of the power system operations manual was also available for the participant to consult at all times during the experiment. This operations manual contains instructions for resolving common contingencies in the grid and evaluating the severity of violations, as well as finding use in training sessions.

The experiment took place over the course of three partial-day sessions. At the beginning of each day, the participant was fitted with the Zephyr bioharness. The first day included a training session, so that the participant could gain exposure to the layout of the room and consoles, had time to work with the IEEE 118 Bus network that was used in the scenarios^3^, and had an opportunity to practice using both the PowerWorld and ACAT software. Additionally, training is assumed to be sufficient enough to dampen learning effects and performance variability associated with unfamiliarity. A tutorial introduced the participant to the naming and location of generators, the topology of the lines, and the overall power distribution in the network, as well as time to read the 118 Bus system manual. The participant was also informed about the data collected, including button presses and hovers, decisions made in the network, and free response answers to interviewer questions.

Following the training session, the participant was presented with scenarios of varying difficulty (described in Section 4.4) and asked to resolve the contingencies in each scenario. Trials alternated between solving a scenario with and without the presence of an ACAT recommendation. Each of the scenarios that we created were tested under both experimental conditions, but the order of the trials was designed so that the participant did not see a scenario twice on the same day. Following each trial, the participant completed a copy of the survey appropriate for the trial condition (see Section 3.3 for details of the differences).

In this study we are particularly interested in how the human responds to and relies on (or not) ACAT recommendations. We only evaluate ACAT recommendations to the extent that it can provide insight for what the human operator does in a given scenario. In cases where ACAT's recommendations were not perceived as appropriate by the participant, we attempt to capture the human's perception of ACAT rather than explaining ACAT's technical process.

### 4.4. Scenarios

For this evaluation, we constructed a collection of scenarios based on the IEEE 118 Bus network. Some of these scenarios were set aside for use only in the training session. Seven scenarios were used in the experimental trials, which we judge to have three levels of difficulty based on expert power system engineer feedback. Readers less familiar with power systems should take away that scenarios were designed with varying difficulties to resolve contingencies without an aid. We include the technical aspects for the interested reader. The scenario numbers are included in the sections that follow for easy reference to the difficulty of each scenario.

E1. **NOA LIGHTLOAD-HIGHVOLT:** An **easy** scenario that is initialized with light load throughout the grid, but the loss of a 138kV line between substations^4^ WMVERN-1 and N.NEWA-1 begins to cause high voltages in the North region because of reactive power injected into the system by shunt capacitors.
E2. **WOA LIGHTLOAD-HIGHVOLT:** An **easy** scenario that is initialized with light load throughout the grid, but the loss of a 345kV line between BEQUIN-1 and BREED-1 begins to cause high voltages in the West region because of an open-ended line.
E3. **WOA TANNRS-SORENS:** An **easy** scenario centered around the large interchange between the North and West regions. The loss of a 345kV line between TANNRS-1 and SORENS-1 causes power imported by the West region to now come exclusively through long 138kV tie lines, with some lines encountering thermal limits.
M4. **SOA KANAWH-CABINC:** A **medium** scenario beginning with low generation in the southeastern portion of the South region. The loss of a 345kV-138kV transformer between KANAWH-1 and CABINC causes an overload in the South region.
M5. **NOA EASTLI-MUSKNG:** A **medium** scenario centered around the large interchange between the North and West regions. The loss of a 345kV line between EASTLI-1 and MUSKNG-1 risks voltage collapse due to power imported by the West region now coming exclusively through long 138kV tie lines.
M6. **WOA OLIVE IROL 2:** A **medium** scenario centered around the large interchange between the North and West regions. The loss of a 345kV-138kV transfomer from OLIVE-1 to OLIVE-2 causes voltage collapse throughout the West region.
H7. **SOA CA SOLUTION FAILURE:** A **hard** scenario centered around the large interchange between the North and South regions. The loss of a 345kV line between EASTLI-1 and MUSKNG-1 could lead to a possible system blackout due to angle instability along the tie lines connecting the West and South regions.

### 4.5. Example Scenario Approach and Resolution

The experiment was designed to replicate the naturalistic decision making processes used by power system operators first documented by Greitzer et al. (2010) and Greitzer and Podmore (2008). The approach used by operators to resolve CA violations is based on their familiarity with the particular power system, their real-time situational awareness, and their knowledge of historical events and usual causes of common problems in the network. Within a control room, operators synthesize numerous inputs from multiple computer displays regarding system frequency, local voltage, power flow direction, breaker statuses, and whether various power plants are online or offline. They use this information in real time to assess impacts of various contingencies, notably verifying that no thermal limits or stability limits will be violated as a result of a particular contingency.

Operators will refer to the guidelines in their operations manual to evaluate the severity of a particular violation, and the required timeline for which the contingency must be mitigated. Table 3 presents one of the tables regarding voltage violation guidelines from the operations manual given to the participant during the experiment.

**Table 3:** Acceptable actions and timeline for simulated post-contingency voltage violations.

**Voltage limit violated**	**Voltage limit value**	**Acceptable control action**	**Time to correct**
Emergency high	1.10 pu / 110%	All effective non-cost actions	Within 30 min
Normal high	1.05 pu / 105%	All effective non-cost actions	N/A
Normal low	0.95 pu / 95%	All effective non-cost actions	N/A
Emergency low	0.92 pu / 92%	All effective non-cost actions **except** load shedding	Within 15 min
Load dump low	0.90 pu / 90%	All effective non-cost actions **including** load shedding if voltage collapse possible	Within 15 min
Post-contingency IROL transfer limit		All effective non-cost actions **including** load shedding if voltage collapse possible	Within 15 min

To resolve CA violations, operators use a sequential decision process that involves projecting the impact of a particular contingency, tracing the root cause of the violation, and identifying available equipment near source of the problem. The decision process typically follows a series of yes/no questions that operators ask themselves while resolving the CA violation.

**Figure 2:**
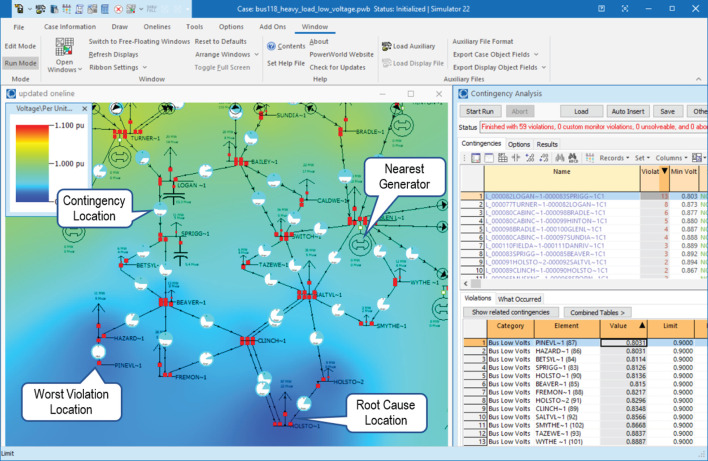
An annotated view of PowerWorld, demonstrating the observations made by an operator seeking to resolve the undervoltage violation described in Section 4.5.

The typical process used by operators for resolving undervoltage violations can be illustrated by tracing a decision tree for a sample violation. A snapshot of the PowerWorld interface for the particular scenario is shown in Figure 2. An operator resolving the particular scenario will observe that the CA results in the right pane of PowerWorld indicates that loss of the LOGAN-SPRIGG transmission line (“Contingency Location” in Figure 2) will result in voltages collapsing to 0.80 per unit (“Worst Violation Location” in Figure 2), which is far below the Load Dump Low threshold given in Table 3. To resolve this contingency, action up to and including load shedding must be taken within 15 min.

The operator will examine the system and identify the source of the problem is the large load at HOLSTO substation without any voltage support (“Root Cause Location” in Figure 2). They will then sequentially ask themselves the questions:

1. **Q:** Under 0.92 per unit (pu)? **A:** Yes.
2. **Q:** Nearby capacitor or reactors? **A:** No.
3. **Q:** Nearby generators? **A:** The closest is at GLEN L substation (“Nearest Generator” in Figure 2).
4. **Q:** Generators online? **A:** No.
5. **Q:** Nearby generators available? **A:** Yes. Request GLEN L generator to start.
6. **Q:** Under 0.92 pu? **A:** Yes.
7. **Q:** Nearby generators: **A:** No.
8. **Q:** Load shedding required? **A:** Yes. Initiate emergency load shed at HOLSTO-1 (“Root Cause Location” in Figure 2).

After completing this decision process in the simulator, the operator will ensure that no new CA violations were created by the solution, and will then proceed to communicate, coordinate, and execute the mitigation actions selected.

In cases where ACAT recommendations are provided to the participant, this process of tracing through the decision flowchart and consulting operation manuals is supplemented by the availability of a proposed solution. The participant was not required to follow this sequence of mitigation steps and can freely choose to ignore the system recommendation. Alternatively, they can explore the recommendation and possibly choose to further refine the solution based on their personal tendencies and knowledge of the system.

## 5. Results

Mirroring the structure of our methodology presented in Section 3, we structure the overarching results of our evaluation of ACAT by first reporting the objective HRV and cognitive load findings in Section 5.1, followed by the performance analysis in Section 5.2. These are followed by our evaluation of the subjective trust data acquired via our surveys (Section 5.3) and a summary of the qualitative feedback collected about ACAT (Section 5.4). In Section 6, we explore the details of individual scenarios and trials to better understand how interactions with ACAT change over time.

### 5.1. Heart Rate Variability

**Figure 3:**
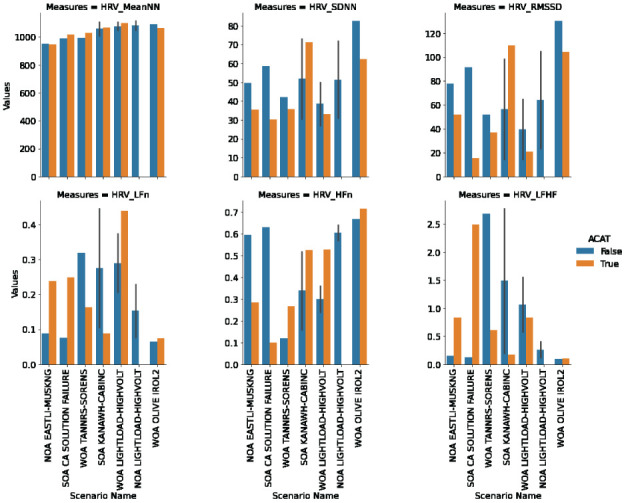
HRV metrics computed for each trial. Descriptions of each metric are provided in Section 3.1.

ECG data was measured to assess cognitive workload experienced by the participant. We included a training session to allow for learning effects and familiarity of working with ACAT to stabilize. Given this training, we assume that cognitive load is a measure of both scenario difficulty and additional burden (or not) of working with ACAT, and not changing over the course of the experiment. As shown in Figure 3, the participant had higher workload when ACAT is present for the scenarios NOA EASTLI-MUSKNG (M5), SOA CA SOLUTION FAILURE (H7), and WOA LIGHTLOAD-HIGHVOLT (E2). Both the time and frequency domain metrics provide evidence in these scenarios. The WOA OLIVE IROL 2 (M6) scenario also provides evidence for higher workload when working with ACAT but only for the time domain metrics. SOA KANAWH-CABINC (M4) represents the only scenario where there is evidence from all metrics that workload increases without ACAT. The remaining scenario, WOA TANNRS-SORENS (E3), has mixed results across the metrics. NOA LIGHTLOAD-HIGHVOLT (E1) was excluded from this analysis because the participant resolved the scenario based on memory and did not allow time to collect enough data for analysis.

**Figure 4:**
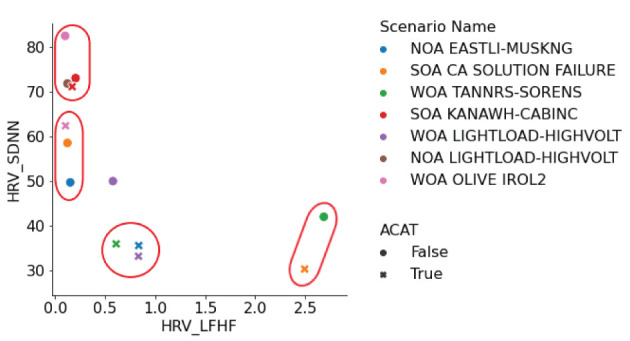
HRV clusters identified in a scatter plot of SDNN vs. LF/HF.

Two metrics were most expressive across the scenarios, SDNN and LF/HF. A manual clustering was performed on the scatter plot of absolute metric values with respect to SDNN (standard deviation of the N-N interval) against LF/HF (ratio of low-frequency power to high-frequency power) to understand where there was corroborating evidence for higher workload among the metrics. Here we included both no ACAT and ACAT trials together. When SDNN is low and LF/HF is high then there is evidence from both metrics that workload is high. We found four clusters in this analysis (Figure 4):

1. **(High SDNN, Low LF/HF)**: WOA OLIVE IROL 2 (M6, No-ACAT), NOA LIGHTLOAD-HIGHVOLT (E1, No-ACAT), SOA KANAWH-CABINC (M4, both conditions)
2. **(Medium SDNN, Low LF/HF)**: SOA CA SOLUTION FAILURE (H7, No-ACAT), WOA OLIVE IROL 2 (M6, ACAT), NOA EASTLI-MUSKNG (M5, No-ACAT)
3. **(Low SDNN, Middle-Low LF/HF)**: WOA TANNRS-SORENS (E3, ACAT), NOA EASTLI-MUSKNG (M5, ACAT), WOA LIGHTLOAD-HIGHVOLT (E2, ACAT)
4. **(Low SDNN, High LF/HF)**: WOA TANNRS-SORENS (E3, No-ACAT), SOA CA SOLUTION FAILURE (H7, ACAT)

The third and fourth clusters indicate higher cognitive load trials than the first two clusters. Noticeably, Cluster 3 only contains ACAT trials and Cluster 4 contains the hard scenario in the ACAT condition. This scatter plot also highlights that trials where ACAT was used (marked with an ‘x' in the figure), corresponding in most cases to either lower SDNN or higher LF/HF.

### 5.2. Performance Scoring

#### 5.2.1. Completion Times

As a measure of performance, we note that completion times tended to decrease throughout the experiment despite changes in difficulty and counter balanced ACAT/No-ACAT trials. This may be an indicator that the participant was still optimizing a strategy for resolving contingencies generally. We note that the participant did not have previous exposure to the 118 bus system, the ACAT tool, or the procedures manual prior to the experiment. The participant did complete a 1-day training session during the first day of the experiment, where he familiarized himself with the ACAT algorithm and 118 bus system.

Completion times cannot be completely explained by learning effects. The scenario, WOA OLIVE IROL 2 (M6), is a scenario where the participant was faster with ACAT although ACAT was used first. In a second scenario, SOA KANAWH-CABINC (M4), the participant was slower with ACAT with this trial being the second time the participant was in this scenario. When scenarios are separated out by difficulty and by ACAT use, there is an ordering where more difficult scenarios required longer completion times (see Table 4). Generally, however, when working with ACAT, our participant was slower at resolving contingencies. A two-way ANOVA did not reveal that neither ACAT nor difficulty was a significant predictor of completion times (see Table 5).

**Table 4:** Completion times for each scenario.

**ACAT status**	**Off**		**On**
**Scenario**	**Original**	**Rerun**	**Original**
NOA EASTLI-MUSKNG	605.0	-	721.0
NOA LIGHTLOAD-HIGHVOLT	591.0	194.0	0.0
SOA CA SOLUTION FAILURE	560.0	-	746.0
SOA KANAWH-CABINC	468.0	150.0	918.0
WOA LIGHTLOAD-HIGHVOLT	462.0	190.0	103.0
WOA OLIVE IROL 2	289.0	-	148.0
WOA TANNRS-SORENS	417.0	-	630.0

**Table 5:** Two Way ANOVA to predict completion time from ACAT and difficulty.

	**Sum sq**	**df**	**F**	**PR(>*F*)**
ACAT	7683.16	1.00	0.16	0.69
Scenario difficulty	334146.65	2.00	3.53	0.06
ACAT: Scenario difficulty	241179.76	2.00	2.54	0.12
Residual	568795.30	12.00	-	-

#### 5.2.2. Action Penalty

**Figure 5:**
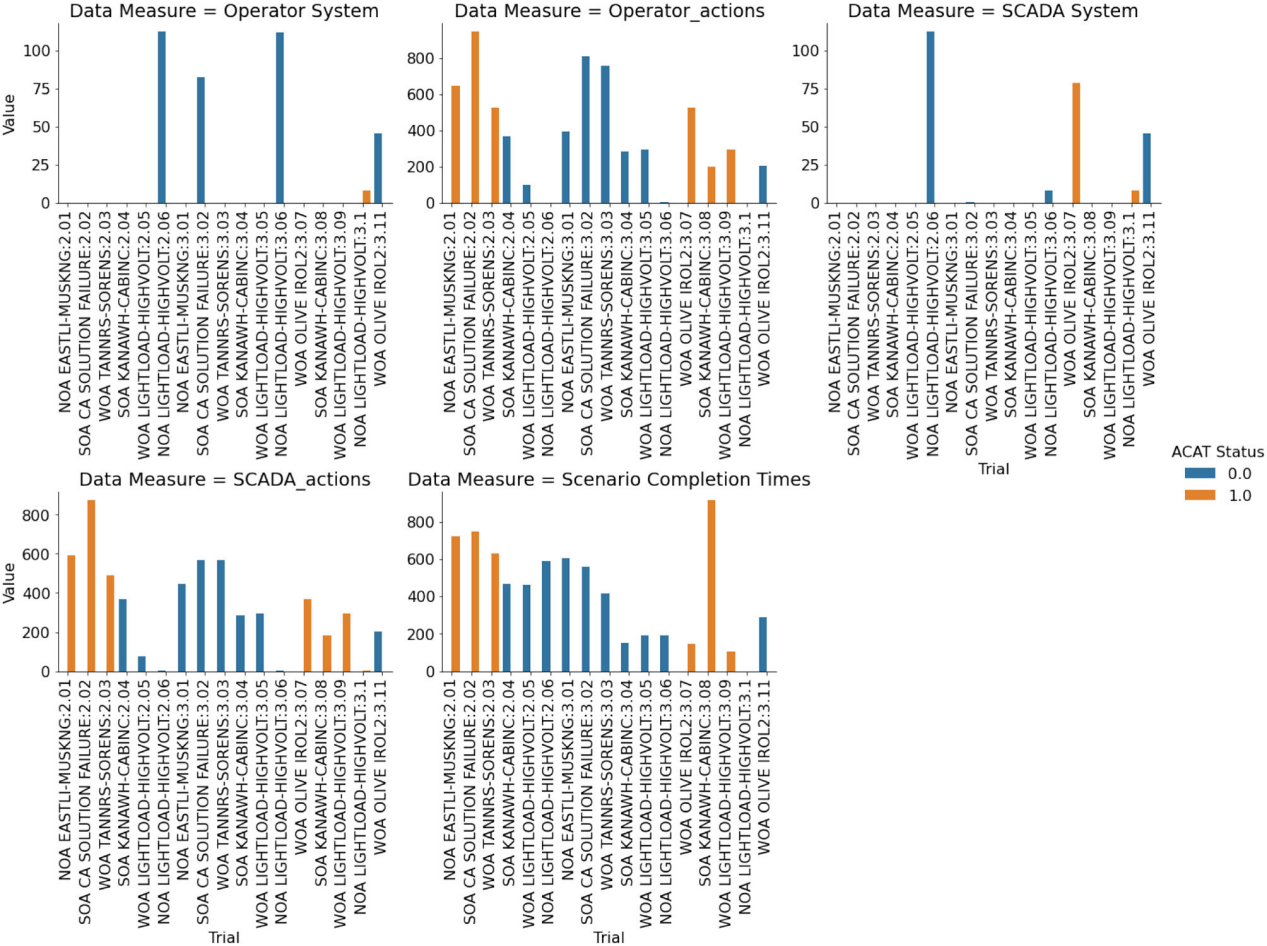
Performance measures computed for each trial.

Action penalties were computed according to the formula provided in Anderson et al. (2022). This metric is a coarse measure of the amount of change the operator inflicts on the system in order to resolve the contingency. This measure is formulated as a penalty function since there is often a direct financial cost associated with re-dispatching generation and inherent risk of equipment failure when opening and closing high voltage circuit breakers. There are two types of data to which action penalties are applied. The first are study-mode data and the second are SCADA console data (see Table 6). As difficulty increases, action penalties also increase. This is true for both the study-mode and SCADA data, with a larger increase from medium to hard in study-mode. Across the two modes, SCADA action penalties are less than study-mode action penalties. Considering ACAT assistance, in four of the scenarios, action penalties increase when ACAT is used. The three exceptions are NOA LIGHTLOAD-HIGHVOLT (E1), SOA KANAWH-CABINC (M4), and WOA TANNRS-SORENS (E3), where in the first, the difference is negligible and in the latter two action penalties decrease (Figure 5).

**Table 6:** Action penalties for each scenario.

**Action type**	**Study-mode actions**			**SCADA actions**		
**ACAT status**	**Off**		**On**	**Off**		**On**
**Scenario**	**Original**	**Rerun**	**Original**	**Original**	**Rerun**	**Original**
NOA EASTLI-MUSKNG	394.08	-	646.84	447.54	-	592.28
NOA LIGHTLOAD-HIGHVOLT	2.29	3.45	1.15	2.29	1.15	1.20
SOA CA SOLUTION FAILURE	809.19	-	947.71	565.31	-	876.23
SOA KANAWH-CABINC	368.90	285.24	200.84	368.93	285.30	182.30
WOA LIGHTLOAD-HIGHVOLT	97.62	296.57	296.57	77.09	295.57	295.57
WOA OLIVE IROL 2	203.43	-	527.92	203.43	-	370.45
WOA TANNRS-SORENS	757.91	-	528.40	567.80	-	491.35

#### 5.2.3. System Penalty

For most of the scenarios, system penalties (Anderson et al., 2022) were zero, which indicates that all contingency violations were resolved and that the operator did not create new violations in a different part of the system. The three scenarios where non-zero values were computed were NOA LIGHTLOAD-HIGHVOLT (E1), WOA OLIVE IROL 2 (M6), and SOA CA SOLUTION FAILURE (H7) (see Table 7). For NOA LIGHTLOAD-HIGHVOLT (E1), SCADA penalty and study-mode penalty without ACAT for the first run matched at 112.69. Similarly the SCADA penalty and study-mode penalty match at 8.41 in the final run with ACAT. However, during the second run (without ACAT), the operator's study-mode system penalty is higher than the SCADA system penalty. The solution on the SCADA console was, in fact, different from the solution on the study-mode console. The participant discovered a solution during this run and rather than implementing on the study-mode console, moved directly to the SCADA console for implementation. The solution of this second run was implemented on the third run where ACAT was available.

**Table 7:** System penalties for each scenario.

**System type**	**Study-mode system**			**SCADA system**		
**ACAT status**	**Off**		**On**	**Off**		**On**
**Run**	**Original**	**Rerun**	**Original**	**Original**	**Rerun**	**Original**
**Scenario**						
NOA EASTLI-MUSKNG	0.00	-	0.00	0.00	-	0.00
NOA LIGHTLOAD-HIGHVOLT	112.69	112.06	8.41	112.69	8.41	8.41
SOA CA SOLUTION FAILURE	82.46	-	0.00	0.88	-	0.00
SOA KANAWH-CABINC	0.00	0.00	0.00	0.00	0.00	0.00
WOA LIGHTLOAD-HIGHVOLT	0.00	0.00	0.00	0.00	0.00	0.00
WOA OLIVE IROL 2	45.63	-	0.00	45.63	-	78.99
WOA TANNRS-SORENS	0.00	-	0.00	0.00	-	0.00

Similarly, in the SOA CA SOLUTION FAILURE (H7) scenario, the participant implemented a better solution on the SCADA console than the study-mode console for the run without ACAT. However, with ACAT the system penalties (SCADA and study-mode) were both zero.

Unexpected penalty results are seen in scenario WOA OLIVE IROL 2 (M6). While the penalties for trials without ACAT are comparable across study-mode and SCADA, the trials with ACAT exhibit a higher penalty for the SCADA solution than the study-mode. In study-mode, the participant resolves the contingency with a penalty of zero, but when switching to the SCADA console, the system penalty increases to 78.99. Upon further examination, the participant did not implement the study-mode solution and instead believed fewer actions (not switching a reactor) would resolve the contingency. The participant had high confidence this single action would resolve the contingency.

### 5.3. Survey Outcomes

**Figure 6:**
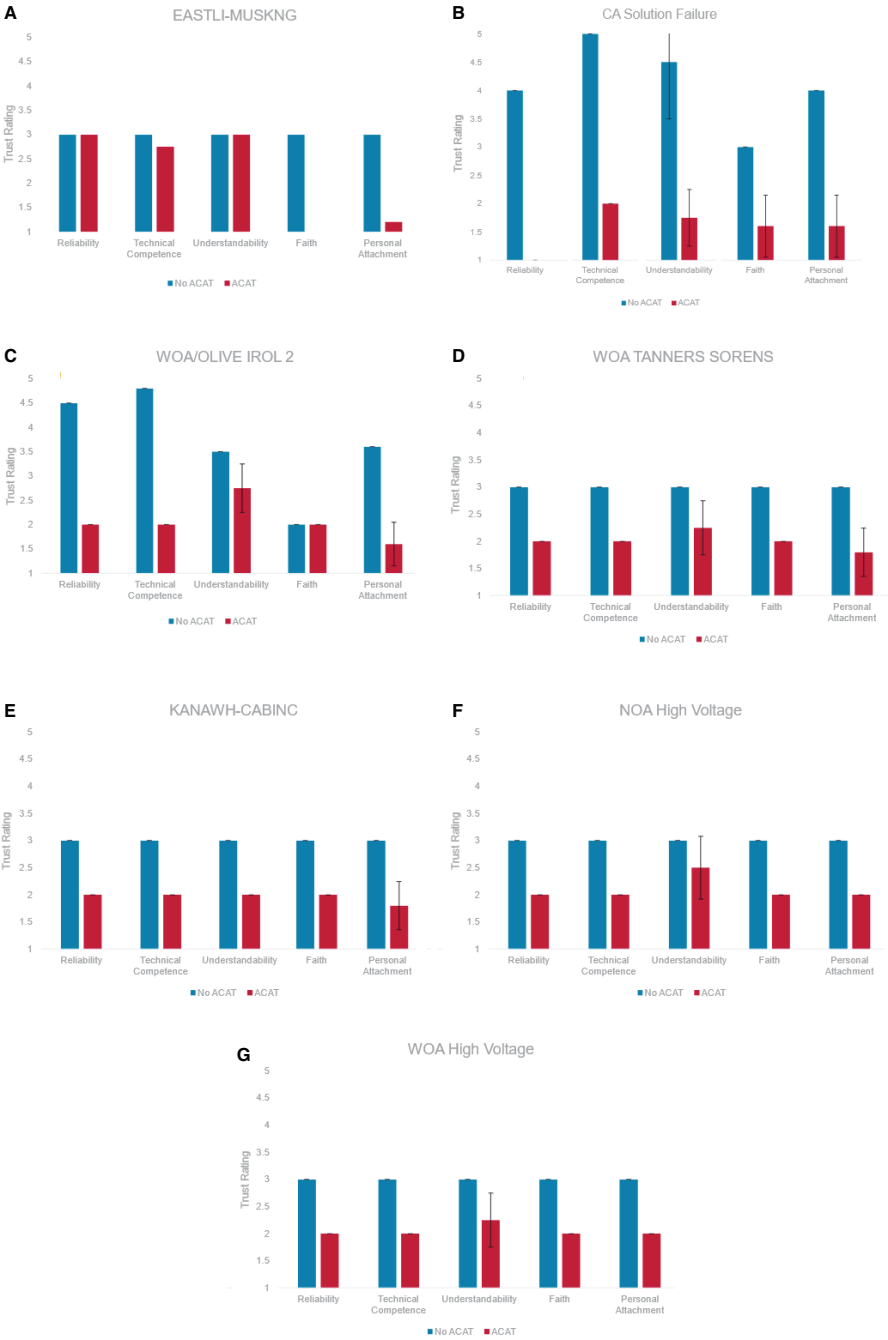
Participant trust levels in both operational procedures (blue) and ACAT (red) for each of the seven scenarios **(A)** NOA EASTLI-MUSKNG, **(B)** SOA CA SOLUTION FAILURE, **(C)** WOA OLIVE IROL 2, **(D)** WOA TANNERS-SORENS, **(E)** SOA KANAWH-CABINC, **(F)** NOA LIGHTLOAD-HIGHVOLT, and **(G)** WOA LIGHTLOAD-HIGHVOLT.

The researchers compared trust in ACAT to the participant's Operations Procedures in seven scenarios. In five of these scenarios, operator trust in ACAT was lower than their trust in Operations Procedures for all constructs. Average trust in ACAT ratings in these five scenarios ranged from *M* = 1.73 (*SD* = 0.31) to M = 2.29 (*SD* = 0.48), one point lower than their in Operations Procedures that ranged from *M* = 3.00 (*SD* = 1.77) to *M* = 3.33 (*SD* = 0.00) (Figure 6).

The only scenarios tested that did not reveal consistently lower trust in ACAT across constructs were NOA EASTLI-MUSKNG (M5) and WOA OLIVE IROL 2 (M6). For the NOA EASTLI-MUSKNG (M5) scenario, Cognitive-Based Trust (i.e., Reliability, Technical Competence, Understandability) in ACAT was comparable to Cognitive-Based Trust in Operations Procedures. In contrast, Emotion-Based Trust (i.e., Faith, Personal Attachment) was considerably lower for ACAT when compared to operations procedures (Figure 6A). When rating trust in ACAT, the construct Understandability showed the greatest variability across all scenarios. A major source of this variability was due to the item “I understand how ACAT will assist me with decisions I have to make” which was consistently rated lower than the other Understandability items. See Table 8 for each Understandability item and its mean rating.

**Table 8:** Understandability items and their associated rating averaged across all scenarios.

**Item #**	**Description**	**ACAT condition mean**
1	I know what will happen the next time I use ACAT because I understand how it behaves.	*M* = 2.57
2	I understand how ACAT will assist me with decisions I have to make.	*M* = 1.86
3	Although I may now know exactly how the AI/ML algorithm works, I know how to use ACAT to make decisions about the problem.	*M* = 2.57
4	It is easy to follow what ACAT does.	*M* = 2.43

For the WOA OLIVE IROL 2 (M6) scenario, trust in the Operation Procedures was higher for all constructs except Faith. Faith in both ACAT and Operation Procedures was low (*M* = 2.00) for this scenario (Figure 6C). Faith in ACAT was comparable to other scenarios. However, Faith in Operation Procedures for was lower for WOA OLIVE IROL 2 (*M* = 2.00) when compared to the average Faith rating across all other scenarios (*M* = 3.00). Survey data did not provide any additional insights into the participant's lack of faith in the procedures during the WOA OLIVE IROL 2 (M6) scenario. The participant did not identify any portions of the procedures that led to a lack of faith during this scenario.

Perceived workload was measured using the NASA TLX. Results revealed very little effect of tool use on workload. The average ratings for Mental Demand, Temporal Demand, Effort and Frustration across all scenarios were lower than average on a 0 to 5 point Likert scale (*M* = 1.29 to *M* = 1.86). Physical demand was non-existent (*M* = 0.00) and perceived performance received the highest possible rating for all scenarios (*M* = 5.00). On average we found very little difference in perceived workload between conditions (Figure 6D). The scenario that showed the greatest difference in perceived workload between the two conditions was SOA KANAWH-CABINC (M4). Mental Demand, Temporal Demand, and Effort were all rated higher when working with ACAT when compared to the No ACAT condition in this scenario (Figure 6E).

### 5.4. Qualitative Feedback

Our debrief captured participant feedback about both the current state of ACAT and the struggles that the participant encountered when evaluating the system recommendations during the experiment trials. Overall, the participant reported that it took more time to interpret ACAT results than it did to implement the solution without ACAT. The participant also identified several usability issues that were not considered by the developers of the tool. Most notably, ACAT used bus numbers rather than substation names when delivering recommendations. Because operators are more familiar with the names, they would encounter additional work in converting the ACAT recommendation into an actionable recommendation. This is not something that operators would have time to do in emergency situations with regulator time constraints.

The recommendations generated by ACAT also differed from common operational practices, in particular recommending shedding load too often (a last resort step for operators) and not increasing power generation often enough. The participant was able to resolve all scenarios without load shedding, despite the ACAT recommendations. The load shedding steps also were not accompanied with other information that an operator would need to know, such as reactive power output. Other common mitigation steps such as line switching and bringing offline power plant units into service were not provided as recommended actions by ACAT.

## 6. Scenario Insights

In this section, we explore some of the individual scenarios, obtaining insights regarding participant interactions with the system under varying scenario conditions and difficulties.

### 6.1. SOA KANAWH-CABINC Scenario (M4)

The SOA KANAWH-CABINC scenario serves as a representative example for a case where the positives of the ACAT recommender outweigh the negatives. In this medium-difficulty scenario, the loss of a transformer has led to some line overloads, low power generation in a substantial region of the grid, and low voltages in some distant buses. Mitigating this contingency requires a participant to redispatch multiple generation units which keeping line thermal lines in mind. This scenario was encountered near the middle of both trial days, and the trial without access to the ACAT recommender was encountered prior to the trial with ACAT assistance.

The HRV data indicate that the participant had approximately the same cognitive workload in both trials, with slightly higher workload during the first trial without ACAT assistance. Although ACAT assistance was provided after the participant had already seen this scenario, the similar cognitive workload in the ACAT condition is backed up in the survey data. The participant showed consistently lower trust in ACAT than in the operational procedures, and the TLX ratings were higher when the participant was working with ACAT. The participant also had a higher completion time in the ACAT condition, and also performed more system checks to confirm the ACAT recommendation. Despite this higher workload, higher effort, and lack of trust in ACAT, the performance results showed that ACAT was helpful, with lower penalties in both study mode and in the SCADA console solution during the ACAT condition.

Overall, this shows that ACAT objectively helped the participant to mitigate the contingency, supporting the technical correctness of the tool. However, the lower trust and higher workload outcomes in the ACAT condition demonstrates the lack of human readiness of the tool. The participant's opinion of the tool had already stabilized in both cognitive and emotional trust constructs, and the assistance of the tool did not affect those opinions.

### 6.2. NOA EASTLI-MUSKNG Scenario (M5)

Following the training sessions, the first scenario encountered by the participant is NOA EASTLI-MUSKNG, a medium-difficulty scenario in which there is a risk of voltage collapse at multiple substations because of power flow across very long tie lines. Mitigating this contingency requires a participant to redispatch multiple generation units while also keeping in mind transfer path limits and power balance. This trial is also performed with access to the ACAT recommender in the first condition, with the second condition run on the next day with only procedures available. This setup provides an opportunity to understand early participant trust in the system.

Indeed, the survey results showed this scenario to be a trust outlier when compared to the other scenarios. The cognitive components of trust were nearly equal between the ACAT and No-ACAT trials (with the values representing trust in ACAT and trust in procedures, respectively), which stands in contrast to the other scenarios in which the No-ACAT trials were consistently higher that the ACAT trials. The emotional components of trust displayed the opposite pattern; these values were minimal in this scenario, with a wide difference between the ACAT and No-ACAT trials. In the other scenarios, the emotional components of trust were still consistently lower for the ACAT trials, but the gap between ACAT and No-ACAT was smaller.

These results show the evolution of trust in the ACAT system, where the cognitive components of trust (reliability, technical competence, understandability) decreased whereas the emotional components of trust (faith, personal attachment) increased across the early trials. Both evolution trends were rapid, with both cognitive and emotional trust in ACAT stabilizing across the later trials (Figure 6).

### 6.3. WOA TANNRS-SORENS Scenario (E3)

WOA TANNRS-SORENS was another of the early scenarios encountered, appearing as the third scenario. This also represents another scenario in which the participant has access to the ACAT recommender first, with the No-ACAT trial encountered on the next day. This scenario is judged to be easy in difficulty, recovering from a loss of a 345 kV line which causes a few line thermal overloads elsewhere in the grid. Mitigating this contingency also requires a small number of redispatched generation units while also keeping in mind the line thermal limits.

The performance results of this scenario show that the action penalties were substantially lower in the ACAT condition than in the No-ACAT condition (Figure 5). This stands in contrast to the other scenarios, in which the gap between ACAT and No-ACAT was either smaller or the No-ACAT condition showed better performance. Despite the better performance with ACAT, the trust levels in the system had already begun to stabilize by this point, with participant trust in the procedures higher than in the ACAT recommendations. This shows that the tool objectively helps the operator to perform better, yet the participant still does not have trust in the ACAT recommendations.

Some indications of this lack of trust are seen in the performance results. While the HRV data shows comparable cognitive load, the participant spent more time mitigating the contingency in the ACAT condition and also performed more system checks to evaluate the quality of the recommendation. The ACAT solution also required more steps to implement, resulting in a higher discrete action penalty score in this condition.

### 6.4. SOA CA SOLUTION FAILURE Scenario (H7)

The SOA CA SOLUTION FAILURE scenario is unique in this collection of experimental trials, both because of its overall difficulty and because the PowerWorld solver was unable to converge to a contingency analysis solution, leaving the participant without that system and therefore fully dependent on the operational procedures and on ACAT when it was available. In this scenario, the loss of a 345 kV line has lead to angle instability in the tie lines connecting the WOA and SOA regions, and the power system is possibly one contingency away from total blackout. Should similar circumstances occur in the real power grid rather than in a simulation, the looming blackout and lack of a contingency analysis tool solution can easily lead to operator panic as they respond to a time-sensitive issue.

The participant encountered the experimental condition with ACAT assistance first and was able to mitigate the contingency. The performance values were roughly the same, with the ACAT trial demonstrating slightly higher action penalties and equal system penalties, but with more system checks performed in the No-ACAT trial. The HRV data was also similar between the two experimental conditions, indicative of the high cognitive load required when exploring and solving this challenge. The TLX survey results were also approximately identical, with only the Effort dimension differing (one point higher for ACAT).

Despite these similarities in workload and performance, the participant rated their trust in the operational procedures much higher than their trust in ACAT across all trust components (Figure 6B). This result is similar to what we detected in other scenarios, with ACAT demonstrating technical assistance but a lack of user trust. It is particularly noteworthy that the ACAT condition of this scenario was the second trial encountered by the participant, immediately after the ACAT NOA EASTLI-MUSKNG (M5) scenario described in Section 6.2. The trust survey results show that the participant's cognitive trust in the ACAT tool dropped quickly, though their emotional trust rose slightly as they had slightly more experience with ACAT.

## 7. Discussion

While ACAT is a low TRL algorithm, there is value in early stage engagement from end users, in this case, from control room operators and engineers. By studying the performance of ACAT with a representative user, we can identify shortcomings in both the technical reliability and human usability of the tool. Overall, we found ACAT to be underperformant across our testing scenarios. In many cases, this underperformance fell in the usability category (as seen in the trust survey results), but there were also technical limitations of ACAT identified in the debrief. Because it required more time to interpret ACAT results than to implement the solution without ACAT, the participant avoided using the system in many of the scenarios where the system recommendations were available. Both technical and usability issues led to participant disuse of the tool in multiple scenarios, where personal expertise and formal operational procedures were preferred for decision-making.

As noted in the overarching results in Section 5, system penalties were typically low in both the ACAT and No-ACAT conditions, indicating contingency mitigation solutions of similar quality in both conditions. This was true regardless of whether the participant encountered the ACAT or No-ACAT trial first. Action penalties presented somewhat more variability in scale, with some trials showing better performance with ACAT and others showing better performance without the recommender.

Results suggest it may not be enough to simply improve system transparency as a technique for building trust. Two key points that the participant identified for improvement are the quality of ACAT recommendations and the interpretability of those recommendations. Because ACAT recommendations did not match common operational procedures and were too aggressive in load shedding, the operator was hesitant to implement those recommendations. Similarly, the challenge of interpreting bus numbers rather than substation names added unnecessary cognitive load to the trials. The identification of these challenges with the system provides actionable feedback for developers working on the next version of ACAT.

The lower initial ACAT emotional trust ratings seen in the NOA EASTLI-MUSKNG (M5) scenario are likely related to its position as the first scenario encountered in the overall experiment. There is some empirical evidence and theoretical support for this explanation. Lowery et al. found the construct Faith lagged behind other constructs as trust in a new system increased with exposure Lowry et al. (2018). In addition, one classic model of trust suggests that Faith is the last component of trust to develop in a relationship (Rempel et al., 1985).

Lastly, the construct Understandability showed the greatest variability across all scenarios. A potential explanation for the lower ACAT condition mean for this item may be the focus on the utility of ACAT in the prompt provided during the experiment. Perhaps the participant is distinguishing between understanding the system (Items 1, 3, and 4) and understanding how the system can provide value to the decision-making task (Item 2).

### 7.1. Limitations

One concern with the design of this study is the introduction of learning effects, in which the participant recalls a previous solution or a previous sequence of operations that are unique to a scenario, therefore enabling a more rapid solution in the second trial with that scenario. Our results showed that while completion times did generally decrease over the course of the experiment, there is no correlation between penalty scores and trial order. Further, we found that many scenarios exhibit different penalty scores, indicative of different strategies being pursued and different solutions being generated in the ACAT and No-ACAT trials.

The participant also reported not feeling comfortable with trusting ACAT recommendations because he did not have sufficient exposure to the system in advance, and so did not have a good understanding of its reliability. Without that comfort, operators will have a tendency to avoid using the tool regardless of its quality. The participant also noted a lack of familiarity with the operational procedures. Since many contingency violations are resolved based on operator knowledge, additional training with both the grid and ACAT are both necessary in future experiments.

## 8. Conclusion

This work presents an evaluation of ACAT, an early TRL recommendation system designed to aid power system operators in their decision-making process for contingency analysis and maintaining an operational power grid. To perform this evaluation, we apply a methodology that captures objective, subjective, and qualitative measures of both technical accuracy and the readiness of human operators to employ the system regularly. These measures enable us to quickly evaluate the impacts of including machine learning into an operator's existing workflow on several dimensions simultaneously. The method supports diagnosing and identifying key areas during the development process for successful integration when deployed into the field. We measure performance and workload in two experimental conditions, one in which the ACAT recommender is available and another in which the operator only has access to fixed procedures.

In addition to compiling overall performance results across all trials, we performed a detailed exploration of individual scenarios to better understand the nuances of how the participant interacted with ACAT. In our experiment, we identified several key issues with the ACAT system, ranging from usability concerns (e.g., recommendations using bus numbers rather than substation names) to mismatches between the system recommendations and current operational procedures (e.g., the frequent occurrence of load shedding in the recommendations). Communicating these technical and usability challenges to developers will further enhance both the TRL and HRL of ACAT in the next release of the tool.

## Data Availability Statement

The raw data supporting the conclusions of this article will be made available by the authors, without undue reservation.

## Ethics Statement

The studies involving human participants were reviewed and approved by Institutional Review Board (IRB) at Pacific Northwest National Laboratory (PNNL). The patients/participants provided their written informed consent to participate in this study.

## Author Contributions

JW, BJ, AA, DC, and CF contributed to the conception and design of the study. AA was on-site during the study and handled much of the data collection. All authors contributed to data analysis, and interpretation, writing to the manuscript, and approved the submitted version.

## Funding

The research described in this paper is part of the MARS Initiative at Pacific Northwest National Laboratory. It was conducted under the Laboratory Directed Research and Development Program at PNNL, a Multiprogram National Laboratory operated by Battelle Memorial Institute for the U.S. Department of Energy under Contract DE-AC05-76RL01830.

## Conflict of Interest

The authors declare that the research was conducted in the absence of any commercial or financial relationships that could be construed as a potential conflict of interest.

## Publisher's Note

All claims expressed in this article are solely those of the authors and do not necessarily represent those of their affiliated organizations, or those of the publisher, the editors and the reviewers. Any product that may be evaluated in this article, or claim that may be made by its manufacturer, is not guaranteed or endorsed by the publisher.

## Footnotes

^1^ https://www.zephyranywhere.com/

^2^ https://www.powerworld.com/

^3^ http://labs.ece.uw.edu/pstca/pf118/pg_tca118bus.htm

^4^The capitalized names within each scenario description refer to substations in the IEEE 118 Bus grid. The scenario names are then formed from combinations of these substation names and conditions associated with the contingencies.
